# Lessons learned from the Amsterdam Cohort Studies among people who use drugs: a historical perspective

**DOI:** 10.1186/s12954-020-00444-6

**Published:** 2021-01-06

**Authors:** Daniela K. van Santen, Roel A. Coutinho, Anneke van den Hoek, Giel van Brussel, Marcel Buster, Maria Prins

**Affiliations:** 1 Department of Infectious Disease, Research and Prevention, Public Health Service Amsterdam, Amsterdam, The Netherlands; 2 Disease Elimination Program, Burnet Institute, Melbourne, Australia; 3 School of Population Health and Preventive Medicine, Monash University, Melbourne, Australia; 4 Julius Centre, University Medical Centre Utrecht, Utrecht, The Netherlands; 5 Department of Infectious Diseases, Amsterdam Infection and Immunity Institute (AI&II), Amsterdam UMC, University of Amsterdam, Amsterdam, The Netherlands; 6 Department of Community and Mental Health Care, Public Health Service Amsterdam, Amsterdam, The Netherlands; 7 Department of Epidemiology, Health Promotion and Care Innovation, Public Health Service Amsterdam, Amsterdam, The Netherlands

**Keywords:** People who inject drugs, Harm reduction programs, The Netherlands, HIV, Hepatitis C, Hepatitis B, Infection, Mortality

## Abstract

The Netherlands is well known for its early adoption of harm reduction (HR) programs at the height of its heroin crisis in the 1970s/1980s, including the implementation of the first needle and syringe program worldwide. In this manuscript, we describe how the Amsterdam Cohort Studies (ACS) among people who use drugs (PWUD) was conceived within the context of the Dutch HR approach, including the challenges scientists faced while establishing this cohort. This required striking a balance between public health and individual benefit, solving research dilemmas in the face of uncertainty, developing controversial innovative and cutting-edge interventions, which changed the prevention landscape for PWUD, and using longitudinal cohort data to provide unique insights. Studies from the ACS covering follow-up between 1985 and 2016 revealed that participation in both opioid agonist therapy and needle and syringe programs led to a major decrease in the risk of HIV and hepatitis B and C infection acquisition. ACS data have shown that the observed decrease in incidence also likely included shifts in drug markets and drug culture over time, selective mortality among those with the highest levels of risk behaviour, demographic changes of the PWUD population, and progression of the HIV and HCV epidemics. Moreover, HR programs in the Netherlands provided services beyond care for drug use, such as social support and welfare services, likely contributing to its success in curbing the HIV and viral hepatitis epidemics, increasing access and retention to HIV and HCV care and ultimately decreases in overdose mortality over time. Given the low coverage of HR programs in certain regions, it is unsurprising that continued HIV and HCV outbreaks occur and that transmission is ongoing in many countries worldwide. If we aim to reach the World Health Organization viral hepatitis and HIV elimination targets in 2030, as well as to improve the life of PWUD beyond infection risk, comprehensive HR programs need to be integrated as a part of prevention services, as in the Netherlands. We should use the evidence generated by longstanding cohorts, including the ACS, as a basis for which implementation and improved coverage of integrated HR services can be achieved for PWUD worldwide.

## Introduction

The Netherlands is well known for its early adoption of harm reduction (HR) programs at the height of its heroin crisis. Both early implementation and broad access to these programs for people who use drugs (PWUD) have been linked to limited transmission of HIV and hepatitis B and C infections in Amsterdam [1]. These findings were based on data from the Amsterdam Cohort Studies (ACS), which had been established in 1985, with follow-up now spanning over three decades, and closed in 2016 [2]. In the ACS, drug use was defined as the use of hard drugs, including heroin, cocaine, amphetamines and methadone. Its contributions included evidence for the impact of HR programs on the risk of blood-borne viral infections, drug use behaviour, all-cause and cause-specific mortality, and HIV and hepatitis C virus (HCV) treatment uptake and adherence. Meanwhile, the ACS has also conducted various multidisciplinary studies, which have increased our understanding of HIV and HCV pathogenesis, viral dynamics and their impact on the immune system [3, 4]. Despite these major contributions, the scientists establishing and leading this cohort were faced with persistent challenges.

In this manuscript, we describe how the ACS among PWUD at the Public Health Service of Amsterdam (PHSA) was conceived within the context of the Dutch harm reduction approach. To this end, we dive into the lessons learned from over 500 published manuscripts and opinion pieces based on the ACS among PWUD and its value to the current epidemiological situation in the Netherlands and other countries. Moreover, given the length of follow-up to date, we take the opportunity to reflect on some of the developments over time to which the cohort was able to provide evidence.

## Background on the origin and early days of the heroin crisis in Amsterdam

The origin of the PWUD population in Amsterdam, The Netherlands, can be traced back to the introduction of heroin to the Amsterdam hippie scene in 1972 [5]. In the early days of the heroin crisis, different groups of people who used heroin emerged in the Netherlands, including Dutch citizens, and German and Surinamese migrants. A few years before Suriname, a former Dutch colony, became independent in 1975, a large group of Surinamese immigrated to the Netherlands [5]. Support and housing for this large new group of Surinamese individuals was lacking, and they had difficulty entering the workforce. These hard social circumstances may have led some to discover heroin, which was more frequently smoked than injected [5]. Around that time, heroin-dependent individuals living in Germany, who were facing stringent punishments for drug use in their home country, started migrating to the Netherlands, where heroin was cheaper and harm reduction policies were put in place. These individuals were referred to as ‘dope refugees’ [6].

In conjunction with the heroin crisis, incidence of sexually transmitted infections (STIs) increased in the Netherlands during this time and was especially high in Amsterdam [7]. Sex with heroin-dependent, female sex workers was a likely source of infection for a significant number of male heterosexual patients diagnosed with an STI—but this group of sex workers was rarely seen at the STI outpatient clinic. PHSA researchers then decided, in 1978, to conduct a small pilot study among 48 female sex workers who used heroin, revealing a very high STI prevalence: 19% had infectious syphilis and 29% gonorrhoea [7]. This prompted the PHSA’s infectious disease department to establish a weekly, out-of-hours (evening) STI outpatient clinic for sex workers who used drugs in 1979 in collaboration with the PHSA’s drug department. This became the first point of contact for further research among PWUD in Amsterdam.

The growing heroin crisis in the 1970s was perceived negatively by most residents of Amsterdam due, in part, to more PWUD in the streets and increasing crime. To manage the crisis, a harm reduction approach, which included the provision of methadone, was adopted and focused on minimizing harm from drug use to PWUD and more broadly to society. While methadone was being prescribed by a few general practitioners and in addiction clinics, not all PWUD were being reached, especially Surinamese PWUD [5]. To facilitate points of contact with the harder to reach, PWUD Surinamese population, a mobile clinic was established in 1979 distributing methadone around Amsterdam [8]. The collaboration between the outpatient STI clinic of sex workers and the PHSA’s drug department facilitated access to methadone for German sex workers who used heroin, as only Dutch nationals or sex workers had been previously allowed to receive methadone.

In the 1980s, it was estimated that around 7000–8000 of the 20,000 heroin-dependent individuals in the Netherlands resided in Amsterdam [9, 10]. Accordingly, the PHSA scaled up their methadone programs in 1981 to three mobile clinics and six outpatient clinics providing different levels of care depending on an individual’s goals: from continued heroin use to abstinence. The mobile clinic was a ‘low-threshold’ HR program, meaning that drug use was allowed when initiating or on methadone, there were no waiting lists and no barriers to enter, exit and re-enter the program. Those motivated to quit drug use could transfer to medium-threshold programs provided at outpatient clinics and by GPs, or to high-threshold programs centred on abstinence provided at addiction care centres. Outpatient PWUD clinics offered a range of health and welfare services provided by medical doctors, social workers and psychiatrists.

In 1983, the main pharmacy located in the city-centre stopped selling low-cost injecting equipment to people who inject drugs (PWID) due to customer complaints. The decreased availability of clean needles and syringes raised concerns about a potential hepatitis B virus (HBV) outbreak among PWID. The Amsterdam’s *‘Junkiebond’* Medical Social Service for Heroin Users *(i.e. Junky Union/MDHG)*, a Dutch organization for PWUD, then promoted the idea to distribute free needles and syringes in Amsterdam [11]. Since this type of service was unprecedented globally, it meant that the PHSA had to develop an approach in uncharted territory. One of the more serious concerns for the PHSA was the public risk of used needles being discarded on the street [11], and thus, the PHSA and the MDHG came to an agreement that used needles and syringes would have to be exchanged for new ones. This borne the first ever needle and syringe programs (NSPs) in 1984 for use in outpatient and mobile clinics and organizations of PWUD [11]. Following its inception, other countries became interested in this approach. The British Secretary for Health visited the PHSA in 1986 and, shortly after, piloted NSPs in several cities in England and Scotland.

### The origin of the Amsterdam Cohort Studies

In 1981, the first case of acquired immunodeficiency syndrome (AIDS) in Amsterdam was diagnosed in a man who has sex with men (MSM) and a burgeoning HIV epidemic then followed [12]. As HIV infection was thought to be concentrated mostly among MSM, the Amsterdam Cohort Studies (ACS) among MSM was established in 1984 at the PHSA [13]. The gay community saw the individual and community level relevance of initiating such a cohort and thus was closely engaged with researchers and actively lobbied to acquire funds for the ACS. At that time, no cases of AIDS had yet to have been diagnosed among PWID in the Netherlands [14]. Meanwhile, emerging reports of cases of AIDS among PWID in the USA incited scientists at the PHSA to propose establishing a cohort study on HIV among PWUD; however, there was little support for such a study.

One of the biggest critics of the cohort was the Amsterdam PWUD organization (i.e. MDHG) [15]. They argued that the study had no individual benefit for PWUD and could have unintended consequences, such as increased stigma. There were also concerns that any proposed cohort would divert focus from other, pressing issues for PWUD and the 12 euros paid to participants for 45 min of study time, which is the estimated remuneration for 45 min of sex work, was perceived as a bribe [15]. In addition, some GPs who prescribed methadone to PWUD were not keen on a cohort study and shared the opinion of the PWUD organization that because NSPs had already been introduced, the spread of HIV would have been unlikely among PWUD in Amsterdam. Researchers would then be obliged to convey the individual and community level benefits of initiating a cohort among PWUD to these stakeholders.

As soon as there was sufficient support from the PHSA’s drug department, it was decided that because of concerns of a looming HIV epidemic in this group, the prospective ACS among PWUD had to be initiated and in 1985 recruitment of the cohort commenced. The overarching original aim of the cohort was to study the prevalence, incidence and risk factors for HIV infection among PWUD. An advisory committee including PWUD organizations was formed to ensure that the MDHG’s criticism was taken into account. HIV testing was performed at each study visit, yet as there was no available treatment, HIV status was only relayed to participants on an opt-in basis. The majority of PWUD participating in the ACS opted to receive their test results [16].

Engagement with the PWUD community at the PWUD/sex-worker outpatient STI clinic and at the mobile methadone clinic facilitated the first wave of recruitment of PWUD to the cohort. Recruitment started at the STI sex-worker clinic and was then extended to methadone outpatient clinics and by word of mouth. As it was hypothesized that sexual networks of injecting and non-injecting PWUD could be mixed and thus an important risk factor for sexual HIV transmission, non-injecting PWUD were also included in the cohort. Inclusion of former, current and non-PWID in the cohort facilitated the study of the incidence of IDU initiation along with the impact of NSP on increases in IDU [17, 18].

Despite the belief that HR programs in Amsterdam would be sufficient in preventing the spread of HIV, the first results of 308 PWUD enrolled in the ACS in 1986 showed that 28% of participants without AIDS-related symptoms had HIV antibodies [2]. Of those testing HIV positive, 97% were current or former PWID and the remaining 3% were male sex workers [2]. The observed HIV prevalence among ACS participants made it clear—PWID were apparently at great risk of infection in the Netherlands—creating a complete paradigm shift of how HR was viewed. Health professionals became much more receptive to the cohort, while HIV prevention materials for this group were rapidly developed and distributed, and NSP in Amsterdam were expanded from 100,000 needles/syringes exchanged in 1985 to 720,000 in 1988 [19].

The cohort continued recruiting at the same locations throughout follow-up except for recruitment at the STI sex worker clinic which stopped in 1997. Then, in 2000 stronger efforts were made to direct recruitment at young PWUD (30 years old or less) because young and recent-onset PWID were found to be at higher risk of HIV [20]. However, follow-up continued for all participants who had been included in the ACS before 2000. This younger PWUD group used cocaine more often than heroin, but with an HIV prevalence at 16% among those who had ever injected, HIV infection was still widespread [21]. Over time, an estimated 15% of the Amsterdam population who injected drugs participated in the ACS [22].

**Fig. 1:**
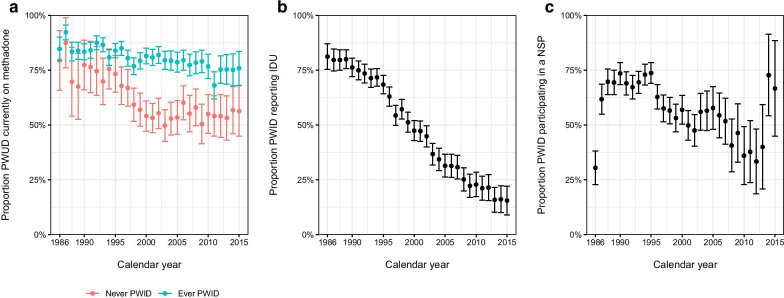
Trends in methadone use, needle and syringe program participation and injection drug use among 1303 PWUD participating in the ACS with at least two cohort visits (1986–2015). PWUD, people who use drugs; PWID, people who inject drugs; NSP, needle and syringe program; ACS, Amsterdam Cohort Studies; IDU, injection drug use. Yearly proportions were calculated by aggregating any report of methadone use (any dose), NSP participation or injection drug use by calendar year among PWUD with a cohort visit in that particular year. Data from 1985 were excluded as there were only 13 records. Data were censored in 2015 (end of data collection). **b** Both current or former PWID only. **c** Lifetime PWID reporting IDU since the previous visit

In 2014, HIV incidence had remained nearly zero for a decade in PWUD. HIV transmission was concentrated in mostly MSM, and pressure was mounting to relocate the total ACS budget to the MSM cohort and expand recruitment in this cohort. Furthermore, the proportion of PWID participating in the ACS who actively injected drugs decreased from 82% in 1985 to 16% in 2015, the uptake of NSP and opioid agonist therapy (OAT) decreased in parallel (Fig. 1), and the median age of participants increased from 27 years in 1985 to 53 years in 2015. In 2016, the cohort officially closed its doors to all participants. At their last visit in 2016, PWUD obtained a small reimbursement for every study visit made (maximum 78 visits). Rumours went around the city about this reimbursement, and after many years of being lost to follow-up, some participants returned to collect this final compensation. Up until the last year, a total of 1,661 PWUD had been included in the ACS of whom 1,303 had at least two cohort visits.

**Fig. 2:**
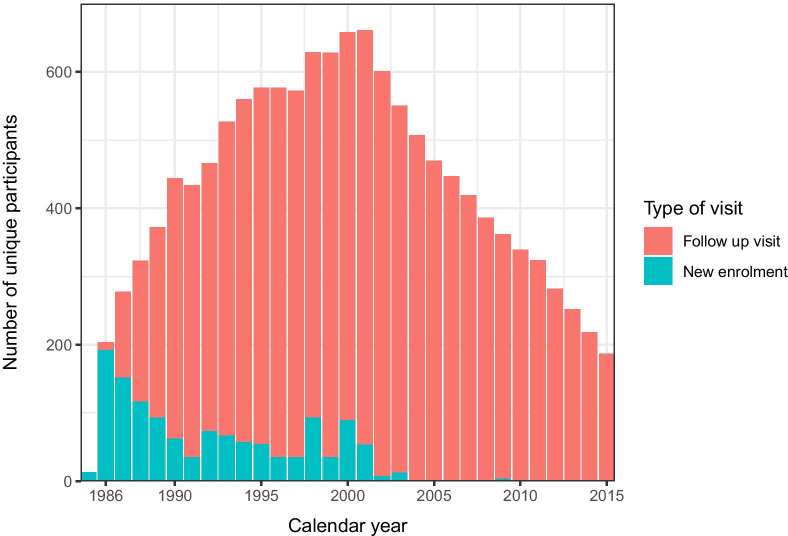
Number newly enrolled and unique follow-up visits between 1985 and 2015 among 1303 with at least two cohort visits

Among these 1,303 participants, the median follow-up in the cohort was 9.4 years (interquartile range (IQR): 3.7–16.6) and the median number of visits was 18 (IQR 7–32). Between 1985 and 2016, 476 participants died and 72 were known to have emigrated outside of the Netherlands. The number of PWUD presenting for a cohort visits fluctuated over the years (Fig. 2). A total of 187 PWUD had a study visit in 2015. The 12-euro reimbursement participants received for each follow-up visit, along with the commitment of study nurses and doctors to provide a flexible and supportive environment for participants, likely contributed to continued participation.

Over the years, HR services other than NSP and OAT have been implemented in the Netherlands. In 1996, the first official drug consumption room (DRC) was launched in Maastricht and as of 2018, 24 DRCs—where mainly heroin and freebase cocaine are used—were operating nationwide [23]. Other unofficial DRCs had been available in Amsterdam in the 1970s and were known as ‘heroin cafés’ [5]. In 1998, the Dutch randomized controlled trial on methadone and heroin co-prescription was initiated, which reported improvements in the physical, mental and social condition of PWUD on heroin co-prescription compared to those on methadone alone [24] and led to heroin becoming a prescribed medication in 2009. Among ACS participants who used heroin at some point between 2009 and 2015, 9% reported ever using heroin co-prescription.

### The legacy of the Amsterdam Cohort Studies among PWUD

Decreased risk behaviour and HR programs likely contributed to a decline in IDU and HIV, HCV and HBV incidence over time [1, 25–27]. However, other factors, such as changes in drug markets, outward migration, demographic shifts within the PWID population and selective HIV-mortality in the 1990s, played an additional role [18, 22]. Several epidemiological and mathematical modelling studies from the ACS have attempted to disentangle the effects of HR and other factors influencing declines in infection incidence and risk behaviour and are summarized below.

### Needle and syringe programs: higher risk behaviour, higher participation

The introduction of NSP among PWUD was more controversial than the provision of methadone, particularly in countries other than the Netherlands. Two Dutch scientists presented the novel NSP approach in 1986 at a conference in the USA at the invitation of American researchers established in this field. At the time, there was still no evidence that NSP could affect risk behaviour or infection transmission. Their presentation sparked heated discussion among participants, including representatives of the African American community who were disproportionally affected by the heroin crisis and were worried that the Dutch approach could bring greater risk of drug use [28]. More specifically, NSP could incentivize non-IDU to IDU, which could outweigh the protective effect against HIV and HBV acquisition, and could be seen as condoning drug use. As a parallel, there was also no evidence that condoms protected against HIV infection at that time, yet programs promoting condom use had been widely implemented across the world.

Early findings from the ACS (1989–1990) demonstrated that HIV-negative PWID who regularly participated in NSP (≥ 90% of needles/syringes coverage by NSP) more frequently engaged in IDU and had a longer history of regular IDU than non-regular NSP participants (< 90% NSP coverage) [29]. This suggested that PWID who have a higher need for needles and syringes are those who access NSP more often. Moreover, this study found that regular NSP participants reported a slightly lower frequency of borrowing needles/syringes (24%) than other PWIDs (33%), but unexpectedly, repeated borrowing only seemed to occur in PWIDs engaging in regular NSP. These findings suggested that borrowing was likely determined by individual characteristics within the regular NSP group and by situational characteristics in other PWIDs. Another early ACS study showed a reduced proportion of needle/syringe borrowing between 1986 and 1992 among current PWID, but this decline could not be ascribed to NSP participation or methadone use [30]. Importantly, ACS data revealed there was no evidence of an increased risk of non-IDU to IDU based on the low and declining IDU initiation rates over calendar time [18].

### Methadone: the importance of receiving the right dose

During the initial planning for methadone distribution around Amsterdam, health professionals were aiming to connect with harder to reach PWUD and these individuals feared that higher dosages of methadone would make them addicted to methadone and heroin would have less of an effect. Consequently, the PHSA provided low doses of methadone to PWUD as part of their low-threshold program. Based on the work by Peter Selwyn in New York, reporting that 60 mg/day was the adequate dose of methadone to promote retention in OAT programs and thus reduce the spread of HIV, the methadone dosing policy changed in the Netherlands in 1991 [31]. In line with these changes, the mean methadone dose among ACS participants increased from 41 mg/day in 1985 to 59 mg/day in 1994. The initial mean low dosing could partly explain why no protective association between methadone use and HIV risk behaviours was observed in an earlier ACS study [32].

To prevent PWUD from receiving multiple doses of methadone via various program levels and prescribers, the central methadone register (CMR) was initiated in 1981 and recorded all methadone prescriptions until 2012. Linking data from this registry with the ACS data, we showed that between 1985 and 1994, the majority of newly enrolled ACS participants (86%) were receiving methadone from low-threshold programs, compared to 10% and 4% from medium- and high-threshold programs, respectively. Importantly, methadone dosages reported from ACS participants were shown to be highly concordant with data from the CMR (weighted kappa = 0.97) [33], while unfortunately today, the validity of self-reported methadone dosages is still oftentimes questioned.

Findings from the ACS demonstrated that higher doses of methadone were associated with lower HIV risk behaviours, including frequency of inconsistent condom use with clients among female sex workers and borrowing of syringe/needles, and an additional methadone dose increase of 5 mg or more per day per year was a predictor of IDU cessation for at least one year [34, 35]. Another ACS study showed that lower methadone dosages (< 60 mg/day) were associated with higher risk of relapse to more frequent heroin use after a cessation episode compared to higher methadone dosages [36]. A later ACS study showed that the prevalence of IDU had exponentially decreased between 1986 and 1998 and that these decreasing trends were not confounded by ‘maturing out’ (i.e. increasing age) [18]. However, prior to 1991, when methadone dosing became adequate, IDU was already declining, suggesting that higher community-wide methadone dosages alone could not explain the continued reductions in IDU trends among ACS participants [35]. Hence, it was hypothesized that ecological factors, such as changes in drug markets and drug use culture, likely played an important role in the decrease of risk behaviour in this group [18].

Heroin, methadone and injecting use patterns are not only highly variable between cohort participants, but also within participants [25]. An ACS study indicated that there were five distinct longitudinal patterns describing injecting trajectories between 1985 and 2005 among PWID [37]. Three of these trajectories displayed stable injecting risk behaviour over time and two displayed a downward trend: a group who decreased injecting early during follow-up (13% of all participants) and another group who showed a gradual decrease over follow-up time (12%). Interestingly, IDU patterns in the ACS were similar to those observed in the USA, suggesting that these injecting trajectories can exist irrespective of cultural differences [37]. Another ACS study reported that among PWUD with a history of addiction to heroin, cocaine and/or amphetamines, abstinence to these drugs and methadone for at least four months was observed in 27% at 20 years from initiating regular drug use [38]. It should be noted, however, that individuals who cease injecting drugs may be more likely to be lost to follow-up, thereby leading to an underestimation of drug cessation in the cohort. While the ACS studies suggested that adequate methadone dosages had a positive impact on drug use cessation, long-term cessation was uncommon, thus consistent with the concept of addiction as a chronic disease.

### Combination of OAT and NSP is key

By the beginning of this millennium, there was individual-level evidence that ACS participants receiving methadone decreased their HIV risk behaviours. Moreover, participants engaging in NSP reported a higher frequency of IDU and a slightly lower frequency of borrowing needles and syringes compared to those who did not [29]. From data outside the ACS, there was conflicting evidence on whether OAT and NSP could decrease individual infection risk [39–41]. Since both OAT and NSP were being used as part of a comprehensive HR program in Amsterdam, ACS researchers decided to investigate the effect of combining both interventions compared to these interventions alone. This resulted in a landmark study from the ACS showing that full participation in HR programs—that is, 100% NSP coverage and at least 60 mg/day of methadone—was associated with a substantially decreased risk of HIV and HCV, acquisition while suboptimal combinations or single use of NSP and OAT was not [42]. These observations were later confirmed by other observational studies outside the Netherlands [43, 44]. This analysis was paramount to understanding why the evidence of a protective effect from individual HR program components on infection risk was either lacking or conflicting.

Nevertheless, there were speculations that the declining trends in HIV and HCV incidence observed among ACS participants were due to alternative explanations, and hence a modelling study using ACS data was initiated to assess several scenarios with and without HR programs. It was hypothesized that, in addition to the effect of HR, PWID with higher levels of IDU risk behaviour may have succumbed to HIV-related disease in the 1980s–1990s (before combination antiretroviral therapy (cART) became widely available) and resulted in a smaller pool of PWID living with HIV and/or HCV. The so-called survivors could have engaged less frequently in risk behaviours and therefore were less likely to transmit these viruses. This study demonstrated that while a decrease in the incidence of these infections was certainly plausible in the absence of HR, the model best performed under the assumption that HR programs gave way to strong decreases in risk behaviour [22]. Building on previous epidemiological studies, this modelling study also highlighted the role of demographic changes (e.g. ageing PWIDs) and natural epidemic progression (e.g. transmission dynamics) as important factors for the observed trends over time [22].

Several reviews of the available evidence on the effect of HR programs and infection risk have been conducted and have found that most studies did not adequately adjust their results for time-varying confounding or the effect of biased selection into HR programs participation (i.e. people engaging in risk behaviours were more likely to participate in HR programs) [18, 22]. As such, definitive conclusions regarding the effect of HR cannot be drawn from previous epidemiological nor mathematical studies. Moreover, as HR programs were implemented before the initiation of the ACS cohort, ACS researchers were unable to compare infection incidence prior to the availability of these interventions. Using data from the ACS between 1985 and 2014, we recently assessed the effect of combined HR program participation on HIV, HCV and HBV infection risk using causal inference methods, which can account for these previously mentioned biases [45]. We showed that the optimal combination of NSP and OAT led to a decrease in risk of 85% for HCV, 44% for HIV and 71% for HBV among PWID participating in the ACS compared to no or partial participation. Using standard statistical methods would have led to attenuated estimates of these protective effects [25].

### Mortality and harm reduction

Each year, information about vital status of ACS participants was obtained by matching the ACS data to the municipal and national population registries in the Netherlands. Causes of death were systematically obtained from hospital records, general practitioners, the national HIV Monitoring Foundation, or coroners. Between 1985 and 1993, nearly 44% of HIV-positive PWID and 12% of their HIV-negative counterparts had died [46]. Early findings from a sample of PWID with and without HIV infection participating in the ACS found no evidence that either methadone use or NSP participation alone were associated with a reduction in all-cause and AIDS-related mortality. However, similar to research on risk behaviours and infection risk, studying the effects of HR on mortality was also prone to bias due to self-selection. More frequent participation in HR programs could be expected among PWID who were deteriorating in health as a result of their drug use or HIV infection and these PWID would have higher risks of mortality. A later study using data up to 1996, during periods when methadone dosing was adequate, found that individuals currently injecting drugs and participating in methadone programs had a lower risk of mortality due to overdose compared to those not receiving methadone [47].

Another ACS study among 1,254 PWUD with at least two cohort visits reported that of the 406 deaths that had been observed between 1985 and 2012, the highest number of deaths (n = 130) occurred during the period 1990–1996. Of these 130 deaths, 40% were HIV-related, 32% due to non-natural deaths (including overdose, accident, suicide and homicide), 21% due to natural causes and 5% due to liver-related disease [48]. Crude all-cause mortality rates have fluctuated over calendar periods: from 28/1000 person-years (PY) in 1990–1996, decreasing to 17/1000 PY in 2001–2005 and rising again to 24/1000 PY in 2006–2012—but crude mortality rates due to non-natural causes continued to significantly decline from 1985 until 2012. When looking at the age- and sex-matched mortality to the general Dutch population, we observed that excess all-cause mortality has continued to significantly decline after 1990–1996 [48]. This strongly indicates that the increase in crude mortality rates in the latest calendar period of the study is likely the result of an ageing PWUD population. Trends in mortality rates are also likely explained by reductions in risk behaviour (Fig. 1), partly driven by HR programs, introduction of cART and ageing PWUD [47–49].

### The indirect benefits of HR programs

Dutch HR programs encompass more than simply the provision of injecting equipment and OAT. These programs form a point of contact with healthcare providers from which PWUD are able to receive services beyond direct care related to drug use. Social workers at HR sites refer individuals to other organizations that can provide stable housing or sheltering and daytime activities. Integrated service centres providing different types of care to PWUD at a single location was officially introduced in 2004 [50]. These services consist of a multi-disciplinary team offering support for social security benefits, help dealing with debt payment/financing, and general and mental healthcare as well as methadone and medical heroin treatment. A holistic approach is important as personal and structural factors, such as homelessness and psychiatric comorbidities, are important predictors of infection risk, barriers to accessing HIV and HCV care and mortality [51].

In 2004, HR programs across Amsterdam facilitated the recruitment of PWUD who actively used drugs, including ACS participants, for an HCV testing and treatment project outside of hospital settings. At the time, HCV treatment duration lasted up to 48 months and could lead to serious psychiatric side effects. Data from this project showed that cohort participants using methadone were more likely to accept HCV testing than those not on methadone and cure rates in this population were comparable to those previously reported among the general, non-drug using HCV-infected population [52]. This was an important finding as most clinicians had believed that continued drug use would likely mitigate cure rates and hence they had refrained from prescribing HCV treatment to PWUD. Another study from the ACS showed that non-adherence to HIV treatment was only reported in 12% of visits of PWUD living with HIV [53], while higher methadone dosages (> 70 mg/day), but not NSP participation, were associated with higher adherence [53].

HR programs do provide advantages not only for the individual, but also for the society as a whole. In a randomized study among heroin-dependent PWUD in the USA, immediate methadone use was shown to lead to decreased number of arrests compared to remaining on a methadone waiting list [54]. The low-threshold availability of methadone from HR programs in the Netherlands may explain the paralleled decline in heroin-related crime and complaints [55]. In addition, these programs have been shown in some settings to be cost-saving in the long term [56].

## Discussion

Written by the pioneers of HR in Amsterdam, those who initiated the ACS and those who witnessed and decided to close the cohort, we recount the story of striking a balance between public health and individual benefit, solving research dilemmas in the face of uncertainty and developing controversial innovative and cutting-edge interventions, which changed the prevention landscape for PWUD. It is evident that since its inception in 1985, the ACS has produced an impressive body of research spanning more than 30 years. Of course, evaluating the causal effectiveness of HR on infection incidence, IDU, morbidity and mortality would be more appropriately assessed through randomized trials. However, the public health urgency of the HIV/HCV epidemic in PWUD made such a trial, with limited follow-up, unethical, impractical and expensive. Longstanding cohort studies, such as the ACS, were needed to provide the necessary evidence of the effectiveness of these programs in addition to unique insights into trends and trajectories in exposures and outcomes over time.

This cohort was initiated in the face of criticisms, teaching us the importance of working together with the community. Over the years, new challenges emerged such as a potential lack of funds to keep running both cohorts, pressure to reallocate the budget and the constant search for funds to continue research using ACS data. One of the most difficult decisions was whether and when to terminate the ACS among PWUD. A decade before the cohort officially ended, there had already been pressure for its closure given that incidence of HIV and HCV was very low. However, the threat of new infection outbreaks and new groups of PWID emerging still persisted and if closed, there would be no possibility to restart the cohort. After ten years of almost zero incident infections, supported by national HIV and HCV notification data, it was then confirmed that this group was at very low risk of transmission. Nevertheless, continued follow-up of cohort participants could have resulted in insights into the ageing PWUD population, trends in direct-acting antiviral uptake and liver-related mortality. However, these research questions substantially deviated from the original aims concerning HIV infection.

Studies from the ACS have taught us that while there is clearly a strong protective effect of HR programs on infection risk and to some degree on mortality risk, it is important to recognize and understand the range of factors contributing to the changing patterns and natural progression of the epidemic. For example, the ageing PWUD population, combined with shifts in drug markets and decreased injecting behaviour, were likely important drivers of declining infection incidence and overdose mortality in Amsterdam. On the contrary, in some countries other than the Netherlands, there has been an increased demand in opioids and new injecting initiates. This, in conjunction with outbreaks of HIV and HCV infection, as has been observed in rural counties in the USA [57], stresses the urgency for implementation and scale-up of HR programs to reduce infection risk.

While problematic heroin use is currently no longer a major public health concern in the Netherlands, the number of prescription opioid users nearly doubled between 2008 and 2017 as well as the number of opioid-related hospital admissions and individuals treated for opioid use disorder [58]. The localized HIV/HCV epidemics among PWUD in the USA were preceded by increased opioid prescriptions. Therefore, vigilance is needed to prevent a similar opioid crisis in the Netherlands as in the USA, although such an event is highly unpredictable. Nevertheless, the lessons drawn from studies among PWUD from the past should remind us to consider the hurdles for future prevention, in particular the difficulty in completely discontinuing opioid use once regular use becomes established. Moreover, as the number of HR services decreases in the Netherlands, the expertise to deal with opiate addiction decreases as well. Therefore, there is always the question as to whether we will be ready to quickly scale up and respond to a potential new opioid crisis in the Netherlands.

Nowadays, the Netherlands has one of the highest prevalence of MDMA consumers and number of producers in Europe [59], while (poly-)drug use, in particular during sex, has increased among MSM in Amsterdam [60]. This brings about a new set of drug-related problems requiring research and novel interventions. Pragmatism remains a cornerstone of the Dutch approach towards drug use beyond the heroin epidemic. For example, testing drug quality/quantity has been easily available since 1992 [61]; although evidence of the effect of drug testing on (non)-fatal overdoses or hospitalizations is lacking, it serves as a means of pharmacovigilance and early warning systems of potentially lethal drugs to the public. Unfortunately, even in countries that have adopted a HR approach towards heroin-dependent PWID, the same arguments of NSPs being a conduit for drug use from the 1980s are being used to discourage the implementation of drug quality testing. Instead, a policy of strong law enforcement and ‘the war on drugs’ approach continues to be applied towards drug use in many countries, without any concrete evidence of its success.

## Unanswered questions

Similar to infection risk, there were significant declines in non-natural deaths (including overdose) observed between 1985 and 2012 among ACS participants, which could be explained by changes in demographic structure, the drug market, selective mortality and broad access to HR programs—but the contribution of each of these factors remains largely unknown. Based on our knowledge of how HR programs reduce the risk of infection, it is important that future studies consider the effect of combination OAT and NSP on mortality alongside each of these two components separately. Moreover, given the rising rates of fentanyl use in some countries—which bears a higher risk of overdose compared to heroin—HR programs might not have the same, previously observed effect on mortality attributed to overdoses. However, to date, it is unknown whether the effect of HR programs on mortality and infection risk differs by the type of opioid used. Regardless, given the degree of bias due to self-selection and time-varying confounding, causal inference methodology should be utilized to the fullest extent possible.

HR programs in the Netherlands offer additional services beyond infection prevention, such as opportunities for stable housing, sexual counselling and access to general and mental healthcare. Therefore, the direct and indirect causal pathways between HR program participation and infection risk lack empirical evidence. For example, lack of stable housing is well known to be associated with increased HIV and HCV acquisition [51] and is associated with decreased HR participation [62]. HR programs may decrease unsafe injecting practices by providing housing and thus safe injecting environments for PWID, further facilitating retention in HR programs and increasing periods of IDU cessation [36]. Disentangling the effects of mediators and direct causes is important for supporting the effects of comprehensive prevention efforts, especially as few HR programs globally are able to offer services other than clean injecting equipment or OAT, or have stricter entry criteria than programs in the Netherlands.

## Conclusion: harm reduction works

Given the low coverage of HR programs in most countries, it is unsurprising that HIV and HCV outbreaks continue to be observed in high-income countries, such as the UK and USA, and that transmission is ongoing in many countries worldwide [57, 63, 64]. Based on data from the ACS, it is evident that HR programs offer opportunities to reach the World Health Organization (WHO) and UNAIDS 2030 targets for HCV and HIV elimination as a public health threat, as well as additional opportunities to improve the overall health and well-being of PWUD. Moreover, as evidenced by the declines in heroin-related crime and complaints over time in the Netherlands, HR programs can offer benefits for society as a whole. We should use the evidence generated by longstanding cohorts, including the ACS, as a basis for which implementation and improved coverage of comprehensive HR services can be achieved for PWUD worldwide.

## Data Availability

Data sharing is not applicable to this article as no datasets were generated or analysed during the current study, except for the production of Fig. 1. The datasets used during the current study to produce Fig. 1 are not publicly available yet due to ongoing data analyses for another study but may be available from the corresponding author on reasonable request, provided that approval is given by the ACS Steering Committee.

## Abbreviations

PWUD: People who use drugs
PWID: People who inject drugs
HIV: Human immunodeficiency virus
HCV: Hepatitis C virus
HBV: Hepatitis B virus
PHSA: Public Health Service of Amsterdam
ACS: Amsterdam Cohort Studies
HR: Harm reduction
NSP: Needle and syringe program
OAT: Opioid agonist therapy
IDU: Injection drug use
MSM: Men who have sex with men
AIDS: Acquired immunodeficiency syndrome
cART: Combined antiretroviral therapy
